# Preference for biological motion is reduced in ASD: implications for clinical trials and the search for biomarkers

**DOI:** 10.1186/s13229-021-00476-0

**Published:** 2021-12-15

**Authors:** L. Mason, F. Shic, T. Falck-Ytter, B. Chakrabarti, T. Charman, E. Loth, J. Tillmann, T. Banaschewski, S. Baron-Cohen, S. Bölte, J. Buitelaar, S. Durston, B. Oranje, A. M. Persico, C. Beckmann, T. Bougeron, F. Dell’Acqua, C. Ecker, C. Moessnang, D. Murphy, M. H. Johnson, E. J. H. Jones, the LEAP Team*, Jumana Ahmad, Sara Ambrosino, Sarah Baumeister, Carsten Bours, Michael Brammer, Daniel Brandeis, Claudia Brogna, Yvette de Bruijn, Chris Chatham, Ineke Cornelissen, Daisy Crawley, Guillaume Dumas, Jessica Faulkner, Vincent Frouin, Pilar Garcés, David Goyard, Lindsay Ham, Joerg Hipp, Rosemary Holt, Meng-Chuan Lai, Xavier Liogier D’ardhuy, Michael V. Lombardo, David J. Lythgoe, René Mandl, Andre Marquand, Maarten Mennes, Andreas Meyer-Lindenberg, Nico Bast, Bethany Oakley, Laurence O’Dwyer, Marianne Oldehinkel, Gahan Pandina, Barbara Ruggeri, Amber Ruigrok, Jessica Sabet, Roberto Sacco, Antonia San José Cáceres, Emily Simonoff, Will Spooren, Roberto Toro, Heike Tost, Jack Waldman, Steve C. R. Williams, Caroline Wooldridge, Marcel P. Zwiers

**Affiliations:** 1 Centre for Brain and Cognitive Development, Birkbeck, University of London, Malet St, London, WC1E 7HX UK; 2 Center for Child Health, Behavior and Development, Seattle Children’s Research Institute, Seattle, WA USA; 3 Department of General Pediatrics, University of Washington School of Medicine, Seattle, WA USA; 4 Department of Computer Science, University of Washington, Seattle, WA USA; 5 Development and Neurodiversity Lab, Department of Psychology, Uppsala University, Uppsala, Sweden; 6 Center of Neurodevelopmental Disorders (KIND), Centre for Psychiatry Research; Department of Women’s and Children’s Health, Karolinska Institutet and Child and Adolescent Psychiatry, Stockholm Health Care Services, Region Stockholm, Stockholm, Sweden; 7 Swedish Collegium for Advanced Study, Uppsala, Sweden; 8 Centre for Autism, School of Psychology and Clinical Language Sciences, University of Reading, Reading, RG6 6AL UK; 9 Institute of Psychiatry, Psychology and Neuroscience, King’s College, London, London, UK; 10 Central Institute of Mental Health, University of Heidelberg, Mannheim, Germany; 11 Department of Psychology, University of Cambridge, Cambridge, UK; 12 Department of Cognitive Neuroscience, Donders Institute for Brain, Cognition and Behaviour, Radboudumc, Nijmegen, The Netherlands; 13 NICHE-Lab, Dept. of Psychiatry, UMC Utrecht Brain Center, Utrecht, The Netherlands; 14 Interdepartmental Program “Autism 0-90”, University of Messina, Messina, Italy; 15 Human Genetics and Cognitive Functions, Institut Pasteur, UMR3571 CNRS, Université de Paris, 75015 Paris, France; 16 Department of Child and Adolescent Psychiatry, University Hospital, Goethe University, Frankfurt am Main, Germany; 17 Department of Psychiatry and Psychotherapy, Central Institute of Mental Health, University of Heidelberg, Mannheim, Germany; 18 Department of Psychology, Ashoka University, Sonipat, India; 19 India Autism Center, Kolkata, India

**Keywords:** Autism, Biological motion, Eye tracking, Development, Biomarker

## Abstract

**Background:**

The neurocognitive mechanisms underlying autism spectrum disorder (ASD) remain unclear. Progress has been largely hampered by small sample sizes, variable age ranges and resulting inconsistent findings. There is a pressing need for large definitive studies to delineate the nature and extent of key case/control differences to direct research towards fruitful areas for future investigation. Here we focus on perception of biological motion, a promising index of social brain function which may be altered in ASD. In a large sample ranging from childhood to adulthood, we assess whether biological motion preference differs in ASD compared to neurotypical participants (NT), how differences are modulated by age and sex and whether they are associated with dimensional variation in concurrent or later symptomatology.

**Methods:**

Eye-tracking data were collected from 486 6-to-30-year-old autistic (*N* = 282) and non-autistic control (*N* = 204) participants whilst they viewed 28 trials pairing biological (BM) and control (non-biological, CTRL) motion. Preference for the biological motion stimulus was calculated as (1) proportion looking time difference (BM-CTRL) and (2) peak look duration difference (BM-CTRL).

**Results:**

The ASD group showed a present but weaker preference for biological motion than the NT group. The nature of the control stimulus modulated preference for biological motion in both groups. Biological motion preference did not vary with age, gender, or concurrent or prospective social communicative skill within the ASD group, although a lack of clear preference for either stimulus was associated with higher social-communicative symptoms at baseline.

**Limitations:**

The paired visual preference we used may underestimate preference for a stimulus in younger and lower IQ individuals. Our ASD group had a lower average IQ by approximately seven points. 18% of our sample was not analysed for various technical and behavioural reasons.

**Conclusions:**

Biological motion preference elicits small-to-medium-sized case–control effects, but individual differences do not strongly relate to core social autism associated symptomatology. We interpret this as an autistic difference (as opposed to a deficit) likely manifest in social brain regions. The extent to which this is an innate difference present from birth and central to the autistic phenotype, or the consequence of a life lived with ASD, is unclear.

**Supplementary Information:**

The online version contains supplementary material available at 10.1186/s13229-021-00476-0.

## Introduction

Autism spectrum disorder (ASD) is a lifelong pervasive developmental condition, characterised by social-communication and interaction difficulties and the presence of restricted and repetitive behaviours [2]. Premature mortality is higher than in the general population (OR 2.56, [18]), and the economic cost of ASD in the USA in 2015 was estimated to be between 0.9% and 2% of US GDP (and growing, [30]). Despite these human and economic costs, effective treatments for the core symptoms of ASD are lacking, with clinical trials and drug development hampered by pronounced phenotypic and pathophysiological heterogeneity and a lack of robust and validated measures to assess symptom severity and track symptom progression. Observational studies may provide insights into the mechanisms that underpin symptom emergence and persistence and thus inform treatment development. However, such studies are often characterised by small sample sizes (the median N in Federici et al.’s [15] meta-analysis of biological motion in ASD was 31.5) and a focus on limited age ranges (Federici et al. categorised studies by age, as either “children”, “adolescents” or “adults” with no studies having a sample that spanned more than one category), leading to inconsistent findings and gaps in the literature. Thus, there is a need for rigorous observational studies with large sample sizes to identify neurocognitive mechanisms that can characterise or stratify the ASD phenotype, and serve as endpoints for use in the development of treatments [31].

Given the (often debilitating) social difficulties seen in ASD, much research has focused on the “social brain” (structures such as the amygdala, the orbital frontal cortex, fusiform gyrus and the posterior superior temporal sulcus), which are hypothesised to be responsible for the initial stages of social information processing [35]. These networks generate representations of the complex kinematics associated with human action and gesture which form the basis for more advanced social cognitive processes such as inferring others’ emotions and intentions. Differences in this early-stage processing of the kinematics associated with human actions may contribute to the socio-communicative difficulties that characterise ASD.

One such function of the social brain that has received attention in ASD is the perception of biological motion, a class of motion pattern generated by the locomotion of another individual. Humans and other social species show a remarkable ability to detect biological motion even in highly impoverished visual displays and demonstrate a preference for biological over non-biological motion [4, 13, 37, 39]. This preference is present at birth [42], indicating the possibility of an evolutionarily ancient “life detector” [20] biasing attention towards conspecifics, scaffolding future social learning and increasing cortical specialisation [11]. A reduction in (or absence of) this bias in ASD may be an early cause of social dysfunction, leading to cascading atypicalities in subsequent social development. Bias towards biological motion may have a similar developmental pattern as bias towards faces, initially served by sub-cortical pathways in infancy [20] and cortical specialisation in older ages, with some eventual overlap in brain regions sensitive to both faces and biological motion (e.g. [14]). The extent to which social experience and expertise maintain these biases is unclear. Particularly in ASD, this may mean that a reduction in the bias towards biological motion reflects fundamentally different cortical network specialisation, or is a consequence of reduced social motivation and experience, or both.

One way to measure biological motion processing is to elicit it with the use of point light displays (PLDs), where the joints between limbs on a human body are replaced with points of light, effectively stripping out all visual cues relating to the body except for posture and motion [19]. Studies using such a design have demonstrated reduced looking times to biological over scrambled and non-biological motion in ASD in toddlers [16, 26, 27] and reduced detection performance in children [3, 5, 34]. Whilst some studies in adults have found no case–control differences (e.g. [32]), two recent meta-analyses find overall reductions in biological motion detection and preference in ASD (Todorova et al. 2019, [15]). Even these meta-analyses, however, disagree on how biological motion perception differs across development, Torodova and colleagues report a reduction in case–control differences as a function of increasing age, whereas Federici et al.’s analysis found no such effect. This inconsistency likely arises from differences in the paradigms used by studies that were, or were not, included in the analyses. Further, there remain few investigations of the degree to which biological motion preference relates to individual differences in ASD symptomatology. This is important to understand whether biomotion preference is relevant to the maintenance of concurrent autism-related difficulties and in turn whether biomotion preference paradigms could provide sensitive measures of prognosis or proxy endpoints in clinical trials. Such effects are challenging to probe in a meta-analysis since the underlying raw data are not commonly available. There thus remains a need for large studies with consistent protocols applied with heterogeneous participants that can examine the presence of case–control differences, their generalisability across ages and functioning levels and their relation to individual differences in concurrent and future symptomatology.

In this paper, we report such an eye-tracking experiment that measured visual preference for biological over non-biological motion in a large (*N* = 486) sample of autistic and neurotypical participants, aged from 6 to 30 years of age. Our aims were to robustly assess (1) the magnitude of case–control differences in biological motion preference (relative to non-biological motion); (2) relationships with ASD symptom severity; and (3) developmental effects across childhood, adolescence and adulthood. We consider these results and their implications for the broader literature on biomotion in ASD.

## Material and methods

### Participants

Eye-tracking data were collected in the context of the EU-AIMS Longitudinal European Autism Project (LEAP; [29]). The LEAP study as a whole involved 453 participants with ASD and 311 non-autistic controls, recruited at six sites across Europe (for a thorough description of the sample and a list of sites, see [10]. Participants with ASD had a clinical diagnosis of ASD (according to DSM-IV/DSM-IV-TR/DSM-5/ICD-10 criteria). Ages between 6 and 30 years old were included. Uncorrected hearing or visual impairments, a history of alcohol and/or substance abuse or dependence in the past year, and the presence of any MRI contraindications (e.g. metal implants, braces, claustrophobia) or absence of informed written consent were exclusion criteria in the ASD group. Exclusion criteria of the TD group were the same, with the additional exclusion of diagnosis of psychiatric disorders, or a T-score ≥ 70 on the self-report (adult) or parent-report form (adolescents and children) of the Social Responsiveness Scale (SRS-2).

For details of exclusion from analyses, see section SM1.1 Data Quality. After exclusions, the sample that provided analysable data for the biological motion task was composed of 282 participants with ASD and 204 non-autistic controls.

#### Assessments

At the time of publication, participants in LEAP had taken part in two assessments, baseline and follow-up, which occurred between 12 and 24 months later. The eye-tracking data we report here were acquired during the baseline assessment. We report associations between it and clinical data concurrently (using clinical data at baseline) and prospectively (using clinical data at follow-up). Of the 486 participants included in the current study at Time 1 (baseline), 365 participants (75.1%) returned for a second visit, on average 16.9 months after the baseline visit (SD = 5.0 months, Range = 7.1–30.0 months). We used follow-up data from the VABS-II Socialisation and Communication domains and the ADOS-2 and SRS-2 (available for *N* = 153 participants, before exclusions based upon eye-tracking data quantity and quality). Descriptive summaries for key clinical and demographic variables at the baseline and follow-up assessments by diagnostic groups are given in Table 1.

**Table 1:** Clinical profile of the sample with biomotion data

	Baseline				Follow-up			
	N	ASD	NT	Comparison	N	ASD	NT	Comparison
Sex		77F, 205 M	58F, 146 M			70F, 182 M	51F, 122 M	
Age (years)	282 ASD 204 NT	17.1 (5.6) 6–31	17.9 (5.6) 6–31	*t*(1484) = − 1.649, *p* = 0.100	213 ASD/152 NT	18.2 (5.5) 8–32	18.5 (5.4) 8–33	*t*(1363) = − 0.568, *p* = 0.571
Full-scale IQ	278 ASD 203 NT	100.0 (18.3) 55–148	107.0 (14.6) 62–142	*t*(1479) = − 4.495, *p* = < 0.001	N/A	N/A	N/A	N/A
ADOS-2 social affect CSS	237 ASD	6.0 (2.6) 1–10	N/A		145 ASD	5.9 (2.6) 1–10	N/A	
ADOS-2 RRB CSS	237 ASD	4.4 (2.7) 1–10	N/A		145 ASD	4.9 (2.7) 1–10	N/A	
SRS-2 T-score (parent)	232 ASD 99 NT	71.7 (11.9) 43–95	46.5 (6.9) 37–71	*t*(1329) = 19.749, *p* = < 0.001	83 ASD/54 NT	73.0 (10.9) 43–90	45.6 (6.8) 37–64	*t*(1135) = 16.529, *p* = < 0.001
Vineland socialisation standard score	233 ASD	71.5 (16.2) 20–119	N/A		171 ASD	76.3 (14.5) 30–111	N/A	
Vineland communication standard score	237 ASD	75.5 (15.4) 21–122	N/A		176 ASD	75.2 (15.0) 21–108	N/A	

Summary values are Mean (Standard Deviation)

### Data acquisition

Eye-tracking data in the LEAP study were acquired at all sites. UCAM, RUNMC and UMCU used a Tobii (Tobii AB, Sweden) T120 eye tracker at a sampling rate of 120 Hz, and KCL, CIMH and UCBM used a Tobii TX-300 at a sampling rate of 300 Hz. Raw eye-tracking data were acquired via the Tobii Gaze Analytics SDK 3.0, processed and saved to disk. Trial onset and offset were associated with the current sample of gaze data and time-stamped in the eye tracker’s time format. When a video was playing, an additional timestamp was recorded every 30 frames, in order to ensure constant synchronisation between stimuli and data.

### Stimulus presentation

The screen on a Tobii T120 eye tracker has a diagonal size of 17″ (34.5 cm × 25.9 cm, 32.1° × 24.4° @ 60 cm viewing distance), a native resolution of 1280 × 1024 pixels, and an aspect ratio of 5:4, whereas the screen on a Tobii TX-300 has a diagonal size of 23″ (58.42 cm × 28.6 cm, 52.0° × 26.8° @ 60 cm), a native resolution of 1920 × 1080 pixels and an aspect ratio of 16:9. To ensure that participants saw equivalently sized stimuli at each site, we presented stimuli full-screen on the T120s and within a “virtual screen” corresponding to a 17″ 5:4 display on the TX-300 s, with black borders around the edge of the screen. Stimuli were therefore drawn with an effective display resolution of 37.1 pixels per cm (0.93° per cm) on the T120s and 32.9 pixels per cm (0.89° per cm) on the TX-300 s. To harmonise the reference frame of the gaze data across sites, those datasets from TX-300 sites were re-referenced to the extent of the virtual screen. Gaze to the black borders around the virtual screen was considered off-screen gaze and removed.

Stimuli were presented on Apple (Apple Inc., USA) Macbook Pro computers, using our custom-written stimulus presentation framework (Task Engine, sites.google.com/site/taskenginedoc/), running in MATLAB using Psychtoolbox 3 [6, 25] and the GStreamer library (gstreamer.freedesktop.org) for video decoding.

### Stimuli

Participants viewed 28 trials, each composed of one silent video each showing two point light walkers (PLDs, see Video SM1 for examples), one on the left and one on the right-hand-side of the screen. All videos were 614 × 582 pixels at 30 frames per second and were scaled to 32.6 cm × 31.0 cm (30.4° × 29.0° @ 60 cm) on screen (1210 × 1150 pixels on the T120; 1071 × 1019 pixels on the TX300). Each PLD was approximately (depending upon the spatial extent of each particular motion) 10.0 cm × 16.2 cm (9.5° × 15.4° @ 60 cm), and the separation between the left-hand and right-hand PLDs was a minimum of 16 cm (15.4° @ 60 cm).

Within each pair of clips, one PLD exhibited biological motion (biomotion condition) and the other non-biological motion (control condition). The biological motion videos were based upon Annaz et al. [3] and included primitive motor, affective, communicative, tool-oriented or goal-oriented movements from the CMU motion capture database [8]. The control motion videos were either: (1) rotated control, in which the PLD was in the same starting posture as the matched biomotion PLD but rotated on the y-axis and used the dominant frequency of hip motion from the human biological motion, as determined by autocorrelation, to define the rotational speed, and (2) scrambled control, in which the light point locations were scrambled but moved with velocity and acceleration profiles matched to the biomotion PLD, phase-scrambled at each point light by a random assignment of phase offsets evenly sampled across the dominant hip movement frequency and random shuffling of point z-coordinates (head-to-heel axis). Embedded conditions included the control type (scrambled or rotational) and the orientation (horizontally flipped or not flipped), as well as the specific content class of the human biological motion: locomotion (11 videos), action (pulling/digging, 4), greeting (4), shaking hands (4) and dancing (5). Stimuli content (i.e. the base human biological motion video) was shown in a fixed order, with control type and orientation counterbalanced across participants.

### Procedure

The biomotion stimuli were presented as one part of an approximately 50-min eye-tracking protocol embedded within a larger battery of experimental tasks and behavioural assessments (see [29], for a comprehensive description). Trials were presented in four blocks of seven trials, which were interspersed amongst other tasks (not reported here). The four blocks of the biological motion task were presented within an approximately 15-min section of the battery, starting after approximately 10 min of stimulus presentation. Five breaks were programmed into the script, one of which occurred before the start of the biological motion task and another of which occurred in the middle after two blocks. Participants could take additional breaks at any point if desired.

At the start of the eye-tracking assessment, the experimenter positioned each participant in front of the eye tracker. Online feedback was given to allow a position to be chosen as close as possible to the centre of the eye tracker head box, to maximise data quality. An automatic five-point calibration was then performed. Participants were told *“I’m now going to show you some pictures and videos on the screen. You don’t have to respond to them, so sit comfortably and watch them as they appear”.* At the beginning of each trial, a gaze-contingent fixation stimulus was presented in the centre of the screen; when gaze fell upon this stimulus, the trial started. If the participant became bored or fussy, the experimenter could skip the current trial and move on to the next. Skipped trials were marked in the data and excluded from analysis. At the end of each trial, the script automatically started the next trials of either the same or a different task.

### Data reduction and metric generation

#### Preprocessing

All data were downsampled to a 60 Hz sampling rate. For gaze samples where binocular information was available (both eyes detected by the eye tracker), we took the arithmetic mean of the left and right eye position. For monocular samples, we used the position of whichever eye was available.

#### AOI scoring and interpolation

Next, we placed an area of interest (AOI) around the location of each of the two PLDs and counted the total number of gaze samples within the biomotion and control PLD AOIs. To minimise the confounding effect of data quality on our measures of Proportion Looking Time and Peak Look Duration, we performed two post-processing steps on the AOI scores (1) interpolation of missing data in AOI scores over gaps of < 200 ms between two identical AOIs and (2) only allowing an AOI to be activated after a minimum number of gaze samples (representing 50 ms) fell inside its area. After post-processing the AOI scores, we then computed two DVs per AOI (biomotion/control) for each trial: Proportion Looking Time (number of samples in AOI/number of samples in both AOIs) and Peak Look Duration (duration of the longest look to that AOI during the trial duration). For more details of these steps, please see SM1.2.

### Clinical measures

#### Autism diagnostic observation schedule-second edition (ADOS-2)

The ADOS-2, a standardised observation assessment for core ASD symptoms, was used to assess current symptoms in ASD participants (Module 1: *n* = 1; Module 2: *n* = 1; Module 3: *n* = 102; Module 4: *n* = 140; missing: *n* = 2). Calibrated severity scores (CSS) for social affect (SA), restricted and repetitive behaviours (RRB) and Overall Total were computed, which provide standardised autism severity measures that account for differences in the modules administered. CSS range from 1 to 10, with higher scores indicating more severe ASD symptom severity.

Internalising and externalising behaviours were measured using the *Development and Well-Being Assessment* (DAWBA; [17]), a semi-structured parent/carer interview designed to generate prediction scores for ICD-10 [44] and DSM-IV-TR [1] psychiatric diagnoses. DAWBA scores reflect six levels of predication (i.e. from ~ 0.1 to > 70%) of the probability of meeting clinically relevant diagnostic criteria for a disorder, ranging from very unlikely (~ 0.1%) to probable (risk score > 70%).

#### Cognitive function

Cognitive function was assessed with either the *Wechsler Abbreviated Scales of Intelligence-Second Edition* (WASI-II), or if unavailable the WISC-III/IV in children and WAIS-III/IV in adults. Standardised estimates of verbal IQ (VIQ), performance IQ (PIQ) and full-scale IQ (FSIQ) with *M* = 100 and SD =  ± 15 are reported.

To standardise data across sites, IQ was prorated from two verbal subtests (vocabulary and similarities) and two performance subtests (matrix reasoning and block design) using an algorithm developed by Sattler [38] that produces an estimated IQ score that is highly correlated (*r* = 0.93) with a full-Scale IQ obtained by administering the complete test. Age-appropriate national population norms for each site were used to derive standardised estimates of an individual’s intellectual functioning. Where recent IQ scores from previous assessments were available (less than 12 months in children; less than 18 months in adolescents and adults), IQ tests were not repeated.

#### Vineland adaptive behavior scale-second edition (VABS-II)

The VABS-II [40] is a semi-structured parent interview that assesses adaptive functioning across three domains in > 6-year-olds: Communication, Socialisation, and Daily Living Skills. For each domain, standard scores have a mean of 100 (SD = 15), with lower scores indicating greater functional impairment, i.e. level of functional skills not commensurate with neurotypical age expectation. Because of our interest in the social domain, we used the standard scores from the Socialisation and Communication domains of the Vineland.

## Autism diagnostic interview-revised (ADI-R)

The ADI-R [36] is a parent interview measure conducted with parents or carers of participants with ASD. Algorithm scores were derived from current and historical symptom presentation to compute relevant total scores for Reciprocal Social Interaction (Social), Communication, and Restricted and Repetitive Behaviours (RRB).

## Social responsiveness scale, second edition (SRS-2)

The SRS-2 [9] is a 65-item questionnaire measure of continuous current autistic traits. The total raw score is transformed into age-adjusted T-scores (and sex-adjusted where applicable). To allow for consistency in the type of rater across the broad age range of the cohort, parent/caregiver-reported scores are used where available.

### Statistical analyses

All statistical analyses were performed in MATLAB R2020b and SPSS 24. For full details of the definition of the linear effects models, please see SM1.5.

#### Data quality and quantity

To understand whether data quality differed between groups, we first counted the number of excluded trials due to (1) low proportion valid samples (< 25%) in a trial; (2) low trial duration (< 4 s); (3) high spatial error (accuracy + precision > 5°). See SM1.1 for full details. For each of these variables, we performed separate linear effects models with a fixed factor of Diagnosis (ASD/NT) and fixed effect of mean-centred Age (in years).

We then focused on valid trials only, and compared the mean (1) proportion valid samples; (2) trial duration; (3) accuracy; and (4) precision between groups. For each variable, we used a separate linear effects model with a fixed factor of Diagnosis (ASD/NT) and fixed effect of mean-centred Age (in years).

#### Presence or absence of biomotion preference

To test for the presence or absence of a biological motion preference, we performed separate one-sample t-tests against zero (representing no preference) for each group, on each DV (Proportion Looking Time, Peak Look Duration).

#### Time on task

The biological motion task was presented within a larger 50-min eye-tracking battery. To investigate whether fatigue affected our results, we investigated how preference for biological motion (as indexed by Proportion Looking Time) and attention to the screen (indexed by Proportion Valid Samples—see 2.8.1) changed as a function of Block number (1–5). We performed two linear mixed models, on Proportion Looking Time and Proportion Valid Samples, with fixed effects Diagnosis and Block.

#### Case–control differences

For each of the biological motion preference DVs (Proportion Looking Time, Peak Look Duration), we performed a linear effects model with fixed independent factors Diagnosis (NT/ASD), Sex (Male/Female) and Site, repeated factor Control Type (Rotated/Scrambled), and mean-centred (across the overall sample) Full-Scale IQ and Age as fixed effects. We examined second-order interactions between Diagnosis and all other factors and effects. We calculated scores on each DV (e.g. proportion looking time) for both the biological and control motion AOIs. To aid interpretability, we transformed each DV from these two separate values (biological/control) to a scalar preference score (biological motion—control motion), with zero indicating no preference, and positive values indicating a preference for biological over control motion.

We used a compound symmetry covariance matrix for the repeated measure. When following up significant interactions in the linear effects model, we used Bonferroni-corrected simple main effects F and t tests. For each of the three data quality metrics, we calculated the number of trials excluded from analysis for each participant. We then performed a linear effects model with fixed factor Diagnosis (ASD/NT) and fixed effect Age in order to ascertain patterns of excluded trials.

#### Associations with ASD symptoms and traits

To investigate associations with symptoms, we examined bivariate and partial (controlling for age) correlations between Proportion Looking Time preference score and both concurrent and change in socially-relevant autism traits within the ASD group only (ADOS-2 Social Affect Calibrated Severity Scores; Vineland Socialisation and Communication Standard Scores; and Social Responsiveness Scale Parent Report t-scores). Because within visual preference tasks a score of zero reflects a lack of preference (compatible with a lack of discrimination), we also tested for the presence of quadratic associations between biological motion preference and the variables listed above (linear models with the clinical variable as the dependent variable, and the linear and quadratic effects of biological motion preference as the predictors).

## Results

### Data quantity and quality

Out of the 593 participants from the LEAP study who were assessed on the biomotion task, 486 (82.0%) contributed valid data. A breakdown of reasons for exclusion from analysis is given in Table 2.

**Table 2:** A breakdown of valid datasets and those excluded from analysis

	*N*	%
Valid	486	82.0
Task not reached in battery	5	0.8
Not acquired (technical fault)	18	3.0
Not acquired (failed to calibrate)	2	0.3
Not acquired (no reason recorded)	34	5.7
Raw data missing	24	4.0
Too few valid trials	24	4.0

#### Comparison of data quality and quantity between diagnostic groups

We performed separate linear effects models on the number of trials excluded due to (1) low proportion valid samples (< 25%) in a trial; (2) low trial duration (< 4 s); (3) high spatial error (see 2.6.1 for full details). The ASD and NT groups did not differ on any of these measures, and nor did Age interact with Diagnosis, all F’s < 2.848, all *p*’s > 0.092. We then performed separate linear effects models on three metrics of data quality for valid trials only, (1) Proportion of Valid Samples; (2) Accuracy; and (3) Precision. The ASD and NT groups did not differ on any of these metrics, and again Age did not interact with Diagnosis, all F’s < 0.843, all *p*’s > 0.359.

### Biomotion preference

#### Proportion looking time

**Fig. 1:**
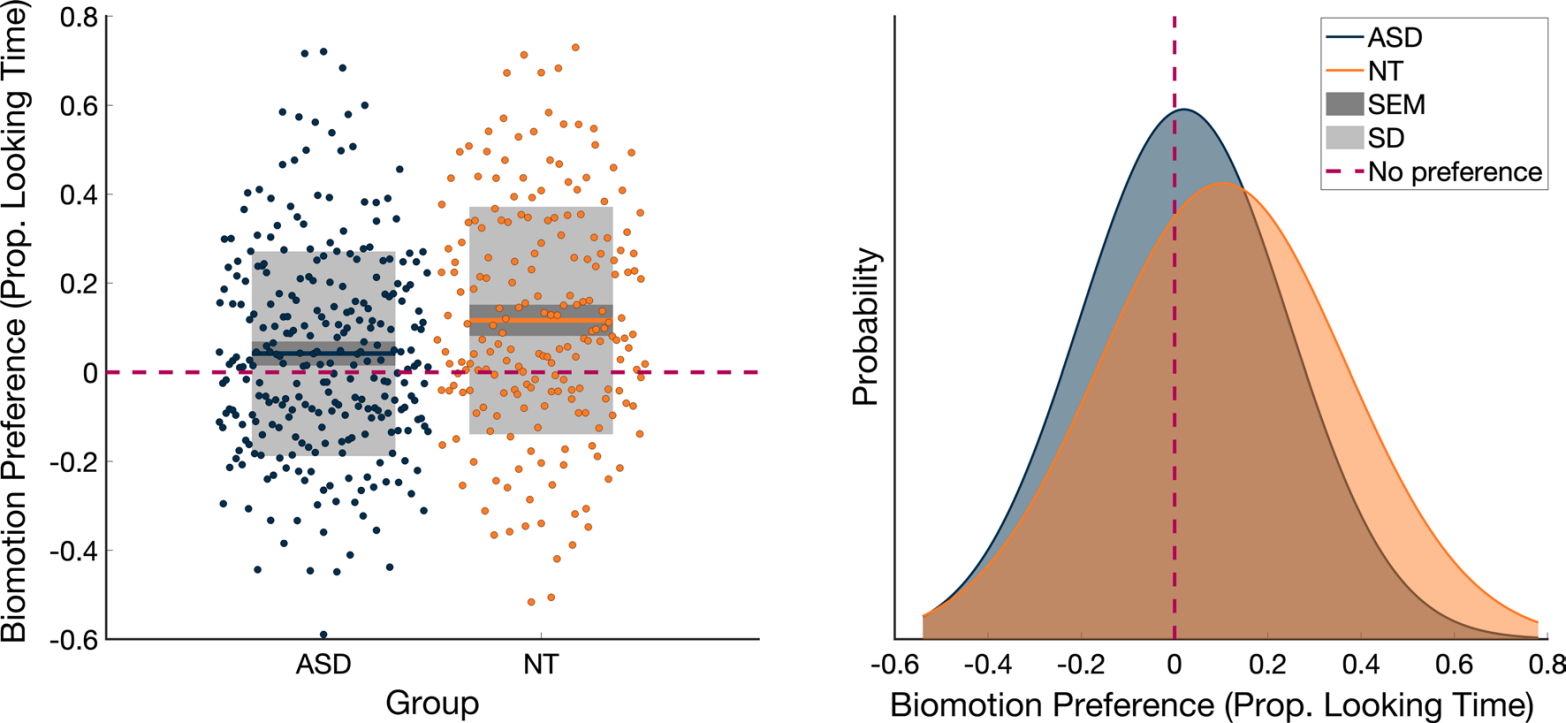
*Top panel* The main effect of diagnostic group on biological motion preference, Proportion Looking Time. A larger preference in the NT than the ASD group. *Bottom panel* The main effect of diagnostic group on biological motion preference, Peak Look Duration (PLD). A longer PLD to biological vs control motion in the NT than the ASD group. *Left panels* individual data, mean, SEM and SD; *Right panels* distribution of biological motion preference by diagnostic group, gaussian smoothed histogram. Dashed zero line represents no preference for biological or control motion

A preference for biological over non-biological motion was present in both groups, *ASD*: *M* = 4.26%, SD = 23.03%, 95% CI = 1.55–7.00%, *t*(278) = 3.096, *p* = 0.002; NT: *M* = 11.65%, SD = 25.72%, 95% CI = 8.10–15.19%, *t*(204) = 6.482, *p* < 0.001. This biological motion preference was significantly smaller in the ASD than in the NT group, *F*(1,466.098) = 9.829, *p* = 0.002, *d* = 0.31 (Fig. 1, top panel).

#### Peak look duration

A similar pattern occurred for Peak Look Duration, with both groups showing a longer look duration towards biomotion (indicated by a positive Peak Look Duration), ASD: *M* = 462 ms, SD = 893 ms, 95% CI = 356 ms-568 ms, *t*(278) = 3.662, *p* < 0.001; NT: *M* = 747 ms, SD = 1037 ms, 95% CI = 603–890 ms. The greater duration of looking at biological motion vs non-biological motion was again smaller in the ASD than the NT group, *F*(1468.904) = 11.097, *p* = 0.001, *d* = 0.30 (Fig. 1, bottom panel).

#### Effect of non-biological motion control type

For both Proportion Looking Time and Peak Look Duration, the preference for biological motion across the whole sample was larger on trials with rotating than scrambled control stimuli (indicating that scrambled motion competed more successfully for attention against biological motion than did rotating motion), *Proportion Looking Time*: *F*(1460.569) = 329.263, *p* < 0.001, *d* = 0.69; *Peak Look Duration*, *F*(1464.164) = 223.922, *p* < 0.001, *d* = 0.59. However, the case–control differences (reported in 4.2.1 and 4.2.2) did not significantly vary by Control Type, both for P*roportion Looking Time*, *F*(1458.747) = 0.021, *p* = 0.884, and for *Peak Look Duration*, *F*(1462.206) < 0.001, *p* > 0.999.

#### Effect of age and IQ on biomotion preference

Across the whole sample, Age did not affect Proportion Looking Time, *F*(1466.781) = 0.117, *p* = 0.732, nor Peak Look Duration, *F*(1469.621) = 0.100, *p* = 0.752. IQ also did not affect either Proportion Looking Time, *F*(1469.142) = 0.098, *p* = 0.755, or Peak Look Duration, *F*(1472.104) = 0.087, *p* = 0.768.

Age did not interact with Diagnosis on Proportion Looking Time, *F*(1458.208) = 0.012, *p* = 0.911 nor on Peak Look Duration, *F*(1461.053) = 0.241, *p* = 0.624. IQ similarly did not interact with Diagnosis on Proportion Looking Time, *F*(1,458.208) = 0.780, *p* = 0.378, nor on Peak Look Duration, *F*(1461.053) = 0.855, *p* = 0.356.

#### Sex differences

At the level of the whole sample (across diagnostic groups), we observed a non-significant effect of Sex on Proportion Looking Time: *F*(1456.008) = 3.629, *p* = 0.057, *d* = 0.019, Females: *M* = 10.7%, SD = 24.9%, 95% CI = 6.5–15%, Males: *M* = 6.0%, SD = 24.0%, 95% CI = 3.5–8.6% and on Peak Look Duration, *F*(1467.245) = 3.519, *p* = 0.061, *d* = 0.014, Female: *M* = 678 ms, SD = 955 ms, 95% CI = 514–842 ms, Male: Mean = 546 ms, SD = 969 ms, 95% CI = 444–649 ms. The case–control effect not did not interact with Sex for either Proportion Looking Time, *F*(1456.008) = 0.015, *p* = 0.903, or for Peak Look Duration, *F*(458.744) = 0.009, *p* = 0.927.

#### Site effects

We did not observe any Site effects across the sample, for Proportion Looking Time, *F*(5466.894) = 0.740, *p* = 0.594, or for Peak Look Duration, *F*(5469.739) = 0.514, *p* = 0.766, nor did we observe an influence of Site on case–control effects for either Proportion Looking Time, *F*(5458.198) = 0.668, *p* = 0.648, or for Peak Look Duration *F*(5461.046) = 0.449, *p* = 0.814.

#### Relationship between Proportion Looking Time and Peak Look Duration

The pattern of case–control effects was the same for Proportion Looking Time and Peak Look Duration. We calculated the bivariate correlation between these two variables, separately for each group. The variables were highly correlated in both groups, ASD: *r* = 0.894, *p* < 0.001; NT: *r* = 0.951, *p* < 0.001.

#### Time on task

Linear mixed models on Proportion Looking Time (indexing biological motion preference) and Proportion Valid Samples (indexing looking to the screen) revealed a change in both variables as a function of Block. Proportion Looking Time increased across blocks, *F*(31,415.887) = 57.471, *p* < 0.001, and Proportion Valid Samples decreased, *F*(31,415.346) = 7.101, *p* < 0.001. There was no interaction between Diagnosis and Block for either Proportion Looking Time, *F*(31,415.887) = 1.656, *p* = 0.175 or Proportion Valid Samples, *F*(31,415.346) = 0.894, *p* = 0.444.

### Association with ASD symptoms and autistic traits

In the ASD group only, we also examined bivariate and age-partialled correlations between Proportion Looking Time and measures of socially relevant autism traits (ADOS-2 Social Affect Calibrated Severity Scores; Vineland Socialisation and Communication Standard Scores; and Social Responsiveness Scale Parent Report t-scores). None survived corrections for multiple comparisons, all *p*’s_corr_ > 0.16. We also examined correlations between Proportion Looking Time and change in ADOS-2, Vineland and SRS-2 scores, between Time 1 and Time 2, taken 12–24 months after participants took part in the biological motion eye-tracking task. None survived corrections for multiple comparisons, all *p*’s_corr_ > 0.18.

**Fig. 2:**
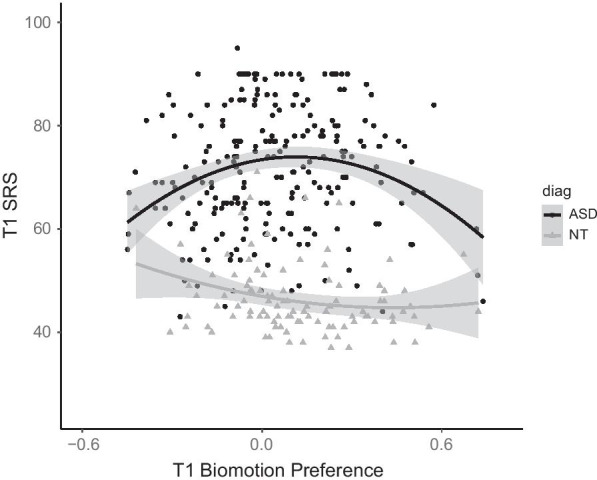
Quadratic association between Biomotion preference and SRS-2 scores within the ASD but not the NT group

Given the nature of the preference task, it may be that social difficulties are indicated by a lack of preference for either the biomotion or control stimulus. We therefore tested for the presence of quadratic relations between biomotion preference scores and the same set of concurrent and prospective clinical phenotypes within the ASD group. The quadratic model was significant for the prediction of concurrent SRS-2 t-scores from biomotion preference, *F*(2227) = 7.39, *p* =  < 0.001 (below the FDR-corrected threshold for eight associations, *ɑ* = 0.006). There was both a significant linear effect, *β* = 9.15, *t*(227) = 2.41, *p* = 0.017, and quadratic effect, *β* =  − 40.09, *t*(227) =  − 3.79, *p* < 0.001, such that a lack of preference (a score in the middle of the scale) was associated with higher SRS-2 scores; the quadratic relation remained significant if age and IQ were included in the model, linear: *t*(227) = 1.34, *p* = 0.18; quadratic: *t*(227) =  − 2.21, *p* = 0.028. The quadratic effect was not present in the NT controls, linear: *t*(97) =  − 2.32, *p* = 0.023; quadratic: *t*(97) = 1.12, *p* = 0.23 (Fig. 2). There were no quadratic associations with the ADOS-2 or Vineland measures either concurrently or prospectively, or change in SRS-2 scores (*F*’s < 3.2, *p*’s > 0.07).

## Discussion

We measured preference for biological motion in a large sample of autistic and neurotypical participants across a wide developmental range, from 6 to 30 years of age. We show a reduction in biological motion preference in ASD, with a small-to-medium effect size. This effect was stable across age (when modelled continuously) and unaffected by IQ and sex differences. Further, within the ASD group a lack of differential preference was associated with higher autistic traits (measured with the SRS-2). Coupled with recent meta-analyses [15, 43], these results should prompt a shift in research emphasis from testing whether group differences in biological motion preference are apparent in autism to studying the cognitive and neural mechanisms that underpin these effects.

It must be noted that not all NT participants showed a preference for biological motion, and that the range of scores in both groups were largely similar (approximately − 0.6 to + 0.8, using Proportion Looking Time). Nevertheless, the mean for both groups showed an overall preference that was greater than zero, and this preference was larger in the NT than ASD group. The magnitude of the biological motion preference across the sample was stable across development. This stability is noteworthy in that it suggests that the attentional bias to biological motion is not merely present in infancy to scaffold socio-communicative learning and development, but rather that it serves an adaptive social purpose throughout the lifespan. Much like face processing, the extent to which biological motion bias is an innate phenomenon “hard coded” into the social brain, or a consequence of exposure to and expertise with social visual information that acts to specialise the social brain for this class of stimulus, is not clear.

Since a preference for orienting to biological motion is present from birth [42], consideration of age-related effects is important. In one meta-analysis, Todorova et al. [43] found larger effect sizes in younger participants’ behavioural performance (although not for eye-tracking measures during passive viewing, where only six studies provided enough information for inclusion in the meta analysis), which they interpreted as evidence of autistic people “catching up” to neurotypical controls through adolescence and into adulthood. However, we observed no significant effect of continuous age between the ASD and NT groups in our eye-tracking data. Our results are therefore most consistent with the presence of a developmentally stable and persistent reduction in preference for biological motion in autism, although this pattern may not be the same for active behaviour as it is for passive viewing.

Both ASD and NT groups showed a larger preference for biological motion stimuli when paired with rotating versus scrambled control stimuli. The motion of the scrambled stimuli was less coherent than that of the rotating stimuli, more chaotic and therefore more information-rich, and unlike the rotating stimuli likely resembled nothing the participants had seen before. We therefore think it probable that this difference reflects a preference for novelty or complexity. Importantly, the magnitude of the case–control differences in biological motion preference was remarkably stable across the two different types of control motion. This suggests that the autistic participants as a group showed no greater or lesser preference for the less coherent, more novel and information-rich motion of the scrambled stimuli than the neurotypical controls. This in turn suggests that the reduction in looking time seen in the ASD group was related to a decreased interest in biological motion per se, rather than an active role of greater attraction to the control stimulus (see [28]), for similar findings in terms of sensitivity thresholds to coherent vs biological motion).

The stability of our results across age, sex and site, and the size of our sample, indicates that a reduction in biological motion preference is a fundamental and likely replicable effect in ASD. The data quality achieved in this multi-site sample was high, and our results are not confounded by differential data quality between groups. We found no differences in missing samples of data, accuracy, precision or number of excluded trials between the ASD and NT groups. This is a demonstration that coordinated eye-tracking data collection across multisite studies is practical whilst maintaining high data quality, even in the face of cultural and linguistic differences in participants and testers, and differences in eye-tracking hardware.

The effect size we report of approximately *d* = 0.30 is smaller than the mean effect size reported by Todorova et al. [43] of *g* = 0.66 and Federici et al. [15] of *d* = 0.60, although these estimates are largely focused on behavioural rather than eye-tracking measures, and the behavioural tasks that participants completed varied. Todorova and colleagues estimated an effect size for the five eye-tracking studies they analysed of *g* = 0.92, although this effect was not significant, and the lower bound of the 95% CIs was close to our observed effect size at *d* = 0.36. The limited number of studies and the relatively restricted age ranges recruited make it difficult to estimate an expected effect size, but on the surface we report here a smaller reduction in biological motion preference in ASD than is found in the extant literature. We speculate that the size and diversity of the LEAP sample (crossing international, linguistic and cultural boundaries) may more accurately reflect the heterogeneity of the autistic population. Smaller, less diverse studies may be effectively sub-sampling this population. Todorova et al. (2020) detected a risk of publication bias in the literature, in the direction of more studies being published with larger effect sizes and large standard errors, and Federici et al. [15] calculated that inclusion of smaller studies estimate to be absent due to publication bias reduced the mean effect size to *d* = 0.47. It may therefore be that smaller studies have produced inconsistent findings, and publication bias has led to only those with significant effect and larger effect sizes being published, thus inflating the mean effect size in the literature.

Another possible explanation for reduced effect sizes is fatigue. A time on task analysis revealed an increase in biological motion preference and a decrease in looking to the screen as a function of experimental block. This latter decrease in attention to the screen in general is likely a result of fatigue; however, the decrease was small (~ 2%) and we believe our quality control procedures (excluding trials with low attention to the screen and interpolating small gaps) were sufficient for this not to threaten the validity of the main findings. It is unclear why biological motion preference increased with time. It may be that the stimuli that the nature of the stimuli presented in later blocks elicited larger preferences, or that by the time participants reached the later blocks they understood the structure of the experiment and spent less time exploring the control stimuli and instead attended more rapidly to the biological motion stimuli. Importantly, we did not find any interaction with Diagnosis for either of these effects, indicating that despite effects of block on the overall sample, the case–control effect remained stable across the experiment.

Once effects have been robustly demonstrated, research to probe the mechanisms that yield them is warranted. Future research using EEG or neuroimaging methods to examine neural correlates of biological motion processing (e.g. [46, 45]) may be important in this regard. Further, longitudinal studies beginning from birth are important to understanding whether these effects could contribute to symptom emergence. Interestingly, one study found that preference for biological motion may reduce between birth and 3 months, when it re-emerged and became stable through toddlerhood [41]. This profile is consistent with other components of social attention (like face and gaze preferences) and may result from a general shift from subcortical to cortical control in the first months of life [21, 23]. Early infancy may be a key window in which to examine the emergence of attenuated preference for biological motion in autism, given that others have observed differing trajectories in other aspects of social attention (e.g. eye gaze) in this period [24]. Overall, the reduction in (as opposed to absence of) a preference in the ASD group suggests an attentional style that down-weights but does not entirely disregard social content. Such a profile was notably not altered in the presence of more severe symptoms of ASD. Our results may thus be better interpreted as an autistic *difference* than an autistic *deficit*—the autistic participants chose differently, not poorly.

### Implications for biomarker discovery, and phenotypic associations

Whilst case–control differences may provide insight into putative mechanisms that could contribute to the aetiology of autism, it is also important to probe the extent to which the case–control differences have utility to other researchers, to industry, and to improving the day-to-day functioning of autistic people. The relatively high distributional overlap between groups and the lack of concurrent or prospective associations with most clinical scales (except concurrently with the SRS-2) suggest that preference for biological motion does not sensitively track core socio-communicative symptoms in ASD, nor is it sensitive to change over time in these symptoms. This challenges the potential utility of measures of passive visual preference biological motion tasks as proxy endpoints for use in clinical trials. Further, the normally distributed preference scores show no indication of the presence of distinct subgroups within the autistic population. Thus, the current data do not provide support for the use of passive visual preference biological motion measures in isolation as stratification biomarkers. Further work should explore whether multivariate methods encompassing a broader battery of social attention measures or measurement at multiple levels (including brain activity) could provide more effective stratification. Alternatively, more complex biological motion tasks that move beyond “first-order” motion detection to instrumental processing of information contained within the biological motion stimulus (e.g. detecting emotion or intention) or the use of temporally (rather than spatially) scrambled control stimuli may be more fruitful [15]. It may also be that atypicality in biological motion preference (a particularly high *or* low score) can serve as a data point within a multivariate composite of other experimental tasks. Such an approach may have promise for the discovery of stratification markers where individuals are grouped according to pattern of scores across a constellation of measures (e.g. [33]).

The lack of clear associations between biological motion preference and dimensional symptomatology measures within the autistic group does not, however, indicate that reduced preference for biological motion is not autism-relevant. It is likely that a variety of heritable causal factors act additively or interactively in early infancy to alter the likelihood of the subsequent development of the behavioural autistic profile [7]. Behavioural symptoms themselves may represent adaptive or compensatory reactions to these early developmental risk factors [22]. Indeed, whilst autism is a highly heritable condition, individual differences in symptom levels within individuals with an autism diagnosis show less evidence of heritability and are instead shaped largely by non-shared environmental factors [12]. When studying neurocognitive phenotypes in individuals with autism, we must thus disentangle those that underpin concurrent symptomatology from those that represent vestiges of early-emerging differences that might have triggered the emergence of behavioural symptoms, but are not related to their maintenance. Given that preference for biological motion is present in neonates [42], that alterations in biological motion preference are apparent in toddlers with ASD [16, 26, 27], biological motion preferences may represent the archaeological trace of a causal factor that acted primarily in early development. Indeed, the quadratic association between lack of preference for biomotion and SRS-2 scores within the ASD group might be consistent with this view. However, such a proposition is not consistent with evidence that 10-month-old infants with later ASD do not show alterations in biological motion preference [16]. Thus, further work is required to understand exactly how and why alterations in biological motion preferences relate to the emergence of ASD.

### Limitations

The paired visual preference task design used here has two related limitations. First, presenting two stimuli simultaneously is more cognitively demanding than serial presentation, in that participants must scan both stimuli first in order to understand what each represents; this may be exacerbated by the relatively visually-sparse nature of PLDs. We might expect younger participants and those with a lower IQ to spend proportionately more time engaged in this initial scan of the stimuli at the expense of time spent expressing a preference. However, we did not find any interaction between visual preference and IQ or age. Second, it is possible that some participants showed a novelty or complexity preference for the control stimuli. This is likely to interact with their preference for biological motion. However, we note that whilst the type of control stimulus (rotating or scrambled) did affect looking times, it did not affect the magnitude of case–control differences.

A two-alternative forced choice (2AFC) paradigm, in which participants must respond as quickly and as accurately as possible to the presence of biological motion in serial presentation, may avoid some of these limitations, although possibly at the cost of disadvantaging the youngest and least cognitively able participants by imposing additional task instructions and requirements.

Despite efforts at recruitment, our NT sample had an average IQ approximately seven points higher than the ASD group, although we believe including IQ as a covariate in our models mitigates this limitation. Our mild-ID (IQ < 70) group was relatively small compared to the majority of the sample without ID, which may limit the representativeness to the autistic population. At follow-up, our quantities of clinical data suffered due to attrition across the sample and challenges in acquiring questionnaire data a second time. Finally, we did not analyse data in approximately 18% of the sample. For 5% of the sample, data were not analysed for reasons that may occur more often in the ASD or mild-ID groups (e.g. too few valid trials), although our statistical comparisons of this data suggested that dropout due to these reasons was as common in the NT as the ASD group.

### Conclusions

This large study robustly demonstrates that there is a reduced (but present) preference for biological motion in children, adolescents and adults with ASD. The lack of dimensional associations with prospective social-communication symptomatology challenges the utility of measures of biomotion as a marker of prognosis or treatment efficacy. However, the clinical profile of autism may result from a common process triggered by a range of underlying factors that leave detectable traces in later development, but have by then become untethered to most surface features of the phenotype [7]. Our data are consistent with the proposal that differences in orienting to biological motion could be a relevant underlying factor, and indicate the importance of pursuing longitudinal studies from infancy of this phenotype.

## Supplementary Information

**Additional file 1: Video SM1:** Illustration of two point-light walker stimuli. **Top:** scrambled control motion and biological motion; **Bottom:** rotated control motion and biological motion. Overlaid heatmaps show average gaze from across the sample.

**Additional file 2:** Supplemental Material.

## Data Availability

The datasets generated and/or analysed during the current study are not publicly available due to an embargo period but are available from the corresponding author on reasonable request.
