# Supplementary material for: Preference for biological motion is reduced in ASD: implications for clinical trials and the search for biomarkers
Source: Mol Autism. 2021 Dec 15;12:74. doi: 10.1186/s13229-021-00476-0 (PMC8672507; doi:10.1186/s13229-021-00476-0)
Supplement: Supplementary file 2 — Additional file 2: Supplemental Material. [file 13229_2021_476_MOESM2_ESM.docx]

**Preference for biological motion is reduced in ASD: implications for clinical trials and the search for biomarkers: Supplemental Material.**

# SM1.1. Data Quality and Quantity

We calculated three metrics of data quality per individual trial:

1. **Proportion of missing gaze samples** (%valid). Trials with %valid < 25% were excluded from further analysis.
2. **Trial duration.** Experimenters could intervene to skip trials in which the participant was distressed or clearly not attending. Trials with a duration < 4s were excluded from further analysis.
3. **Spatial error.** Accuracy and precision were calculated during the gaze-contingent fixation stimulus that preceded each trial. Because the fixation stimulus was always at a known location, and because the trial would not begin until that location was fixated, we can use it to calculate the spatial error between the true gaze location and the gaze location reported by the eye tracker. Accuracy was calculated as the root-mean-square (RMS) of the euclidean distance between the location of each gaze sample and the location of the fixation stimulus. Precision was calculated as the RMS of the euclidean distance between each gaze sample and the centroid of all gaze samples. The separation between biomotion and control AOIs was 6.9° in the horizontal axis. In order to ensure that even a substantially poor calibration (with attendant high accuracy drift or very low precision) would not trigger a non-fixated AOI, we excluded all trials where accuracy + precision was >5° (on the basis that the worst-case scenario on any given trial is for poor accuracy and poor precision to combine such that an individual sample of eye tracking data had measurement error equal to their sum *in the same direction*).

Participants with < 10 valid (out of a total of 28 possible) trials were excluded entirely from the analysis.

# SM1.2. Postprocessing of AOI scores:

1. **Interpolation of AOI scores:** contiguous runs of gaze data samples may be missing for several reasons. Blinks and looks away from the screen generally give rise to relatively long (>100ms) runs. “Flicker”, in which the eye tracker rapidly moves between detecting and not detecting the eyes (often on a sample-by-sample basis) gives rise to very short (1 sample, <10ms) runs. Finally, participant head motion can cause the eye tracker to fail to detect the eyes. Run length here is variable, dependent upon the behaviour of the participant, and on individual differences in how well the eye tracker can detect a particular participant’s eyes. All of these categories of missing data serve to cut contiguous looks to an AOI into several shorter looks, leading to a looking time x data quality confound in that worse data quality gives rise to erroneously shorter look durations. Given that differential data quality between ASD and NT participants is likely, we wish to minimise this confound before analysis. One solution is to interpolate the gaze data itself, but this leads to physiologically impossible patterns where missing data crosses fixations, since linear interpolation will join the two fixation locations with a straight line (as opposed to the expected “square edge” pattern of fixations and saccades). Instead we employed an approach whereby each sample of gaze is converted into a logical vector of “AOI scores”, marked as 1 when gaze fell inside the AOI for a particular sample, and 0 where it did not. We then “interpolate” (or, more accurately, fill in) samples in this vector where the gaze data was missing. To ensure that we do not erroneously assign missing data to one AOI when in fact gaze during the missing period moved to another AOI, we only do this for a) runs of missing data where the valid data on either side of the missing run was to the same AOI, and b) runs with a duration <200ms^^[[1]](#footnote-1)^^.
2. **AOI trigger tolerance**: In cases where precision is low (which are likely to be transitory, occurring after a run of missing data when the eye tracker “recovers” the missing eyes - see Hessels, Corneliseen, Kemner & Hooge, 2015), a stray sample of gaze data may be reported by the eye tracker at some spatial distance from the true gaze location. To avoid triggering the AOI in these circumstances, we apply a “trigger tolerance” of 50ms, meaning that a minimum of 50ms of gaze data must be accumulated in an AOI before the onset of a look is counted.

After post-processing the AOI scores, we then computed two DVs per AOI (biomotion/control) for each trial: Proportion Looking Time (number of samples in AOI / number of samples in both AOIs), and Peak Look Duration (duration of the longest look to that AOI during the trial duration).

We then averaged Proportion Looking Time and Peak Look Duration across all trials for each of the two contrasts (biomotion vs rotated; biomotion vs scrambled). Finally we converted all DVs to a difference score representing biomotion preference (e.g. for Proportion Looking Time we calculated %biomotion - %control). The sign of this variable indicates whether the preference was for biological motion (positive) or control motion (negative), and the magnitude indicates the size of the preference (e.g. for Peak Look Duration, +200ms indicates a preference for biological motion, with peak looks being 200ms longer to the biological than control motion). As such, all reported statistical analyses, means and confidence intervals indicate the effect of independent variables on a relative *preference* for biological motion.

# SM1.3 Means and Standard Deviations for Data Quality and Quantity

In section 4.1.1 we report between-group comparisons of data quantity and quality. In Table SM1 we report the means and standard deviations for these measures.

|  | **Diagnosis** | |
| --- | --- | --- |
| **Measure** | **ASD** | **NT** |
| **Number of trials with Proportion Valid Samples > .25** | 27.12 (2.24) | 27.19 (2.18) |
| **Number of trials with a valid duration (>4s)** | 27.76 (0.65) | 27.81 (0.63) |
| **Number of trials with valid spatial error (Accuracy + Precision < 5°)** | 23.50 (3.98) | 23.34 (4.03) |
| **Mean Accuracy (°)** | 1.60 (0.81) | 1.53 (0.80) |
| **Mean Precision (°)** | 0.30 (0.24) | 0.29 (0.19) |
| **Proportion Valid Samples** | 0.89 (0.13) | 0.90 (0.13) |

*Table SM1. Mean (SD) for data quantity and quality measures, by Diagnosis.*

# SM1.4. Rationale for Selection of Dependent Variables

We selected two dependent variables to measure biological motion preference. Proportion Looking Time indexes the amount of time spent looking at the stimulus, normalised for the amount of time that valid samples of eye tracking data (when the participant was looking at the screen, and the eye tracker was able to detect their eyes). Peak Look Duration is a related, but potentially different, measure. It refers to the duration of the longest “look” (visit) to an AOI over the course of a trial. For example, a ten-second trial with a Proportion Looking Time of 0.5 (50%) could be composed of two five-second looks, or ten half-second looks. These are likely two quite different search behaviours, but only Peak Look Duration can discern one from another.

We did not analyse the AOI that received the first fixation after trial onset for two reasons, 1) the size of the stimuli and their separation from the centre of the screen was such that they did not fall into the fovea at the start of the trial, and 2) the motion of the stimuli require some amount of presentation time before it is possible to discriminate between biological and control motion. For these two reasons, we do not believe it is plausible for there to be a “popout” effect that might guide the first fixation. In other words, at the time of the first fixation, it is unlikely that participants were yet able to determine what they were looking at.

# SM1.5 Definition of Linear Mixed Models

Linear mixed models were performed using the MIXED command in SPSS24. Categorical independent variables were defined as fixed (e.g. Diagnosis) or repeated (e.g. Control Type) factors, and continuous variables (e.g. Age) as fixed effects. We added all main effects and second-order interactions with Diagnosis to the models. For example:

*Proportion Looking Time = Diagnosis + Control Type + Sex + Site + FSIQ + age + Diagnosis*Control Type + Diagnosis*Sex + Diagnosis*Site + Diagnosis*FSIQ + Diagnosis*Age*

We used a compound symmetry covariance matrix and restricted maximum likelihood estimation.

# SM2.1 Stimulus Videos

Video SM1 shows example stimuli from two trials, with gaze heatmap overlaid. Top panel: scrambled control stimulus, Bottom panel: rotating control stimulus.

1. 200ms on the basis that it is not feasible for the participant to begin on AOI 1, then during the period of missing data, make a saccade to AOI 2, remain for a short fixation, and then saccade back to AOI 1. This would represent us entirely missing one look to AOI 2, and to erroneously classify the missing data as remaining on AOI 1 for the duration of the run. [↑](#footnote-ref-1)
